# Supplementary material for: Multi‐dataset identification of innovative feature genes and molecular mechanisms in keratoconus
Source: J Cell Mol Med. 2024 Sep 19;28(18):e70079. doi: 10.1111/jcmm.70079 (PMC11412914; doi:10.1111/jcmm.70079)
Supplement: Supplementary file 2 — Material S2. [file JCMM-28-e70079-s002.zip › 7.GSEA analysis/1.ARL11/KEGG_BUTANOATE_METABOLISM.html]

Details for gene set KEGG\_BUTANOATE\_METABOLISM[GSEA]

|  || Dataset | ARL11.group.cls#h\_versus\_l |
| Phenotype | group.cls#h\_versus\_l |
| Upregulated in class | l |
| GeneSet | KEGG\_BUTANOATE\_METABOLISM |
| Enrichment Score (ES) | -0.3439473 |
| Normalized Enrichment Score (NES) | -1.003498 |
| Nominal p-value | 0.474 |
| FDR q-value | 1.0 |
| FWER p-Value | 1.0 |
Table: GSEA Results Summary

  

Fig 1: Enrichment plot: KEGG\_BUTANOATE\_METABOLISM      
 Profile of the Running ES Score & Positions of GeneSet Members on the Rank Ordered List

  

| SYMBOL | TITLE | RANK IN GENE LIST | RANK METRIC SCORE | RUNNING ES | CORE ENRICHMENT || 1 | GAD1 | na | 1955 | 0.300 | 0.0350 | No |
| 2 | ACSM1 | na | 3283 | 0.228 | 0.0663 | No |
| 3 | ACSM2A | na | 5967 | 0.138 | 0.0310 | No |
| 4 | HMGCS2 | na | 6474 | 0.125 | 0.0545 | No |
| 5 | HMGCL | na | 9012 | 0.075 | 0.0041 | No |
| 6 | L2HGDH | na | 10878 | 0.049 | -0.0347 | No |
| 7 | ALDH7A1 | na | 10913 | 0.048 | -0.0210 | No |
| 8 | BDH1 | na | 11393 | 0.043 | -0.0217 | No |
| 9 | ACSM5 | na | 11426 | 0.042 | -0.0097 | No |
| 10 | AACS | na | 12223 | 0.034 | -0.0222 | No |
| 11 | PDHA2 | na | 12632 | 0.030 | -0.0248 | No |
| 12 | ALDH3A2 | na | 13283 | 0.025 | -0.0360 | No |
| 13 | GAD2 | na | 15039 | 0.011 | -0.0833 | No |
| 14 | HADH | na | 15258 | 0.009 | -0.0867 | No |
| 15 | PDHB | na | 15297 | 0.009 | -0.0851 | No |
| 16 | HMGCS1 | na | 15538 | 0.007 | -0.0898 | No |
| 17 | PDHA1 | na | 17379 | -0.006 | -0.1410 | No |
| 18 | ABAT | na | 17429 | -0.006 | -0.1405 | No |
| 19 | ECHS1 | na | 17627 | -0.008 | -0.1438 | No |
| 20 | OXCT1 | na | 17951 | -0.010 | -0.1501 | No |
| 21 | HADHA | na | 19491 | -0.020 | -0.1883 | No |
| 22 | ACAT2 | na | 20041 | -0.024 | -0.1968 | No |
| 23 | ACSM3 | na | 20910 | -0.031 | -0.2125 | No |
| 24 | ACAT1 | na | 22983 | -0.050 | -0.2570 | No |
| 25 | EHHADH | na | 24876 | -0.074 | -0.2889 | No |
| 26 | ALDH1B1 | na | 25126 | -0.078 | -0.2723 | No |
| 27 | ALDH9A1 | na | 25157 | -0.078 | -0.2493 | No |
| 28 | ALDH5A1 | na | 25959 | -0.090 | -0.2451 | No |
| 29 | ACADS | na | 29390 | -0.169 | -0.2925 | Yes |
| 30 | ALDH2 | na | 31150 | -0.226 | -0.2743 | Yes |
| 31 | BDH2 | na | 31653 | -0.246 | -0.2138 | Yes |
| 32 | OXCT2 | na | 32362 | -0.279 | -0.1492 | Yes |
| 33 | ACSM4 | na | 33324 | -0.346 | -0.0714 | Yes |
| 34 | AKR1B10 | na | 33571 | -0.367 | 0.0333 | Yes |
Table: GSEA details [plain text format]

  

Fig 2: KEGG\_BUTANOATE\_METABOLISM      
 Blue-Pink O' Gram in the Space of the Analyzed GeneSet

  

Fig 3: KEGG\_BUTANOATE\_METABOLISM: Random ES distribution      
 Gene set null distribution of ES for **KEGG\_BUTANOATE\_METABOLISM**
